# The effects of a special sequential mixed-mode design, and reminders, on panellists’ participation in a probability-based panel study

**DOI:** 10.1007/s11135-021-01126-6

**Published:** 2021-03-07

**Authors:** Rolf Becker

**Affiliations:** Department of Sociology of Education, University of Bern, Fabrikstrasse 8, CH–3012 Bern, Switzerland

**Keywords:** Sequential mixed-mode design, Push-to-web design, CATI, Reminder, Competing risks, Event history analysis

## Abstract

The sequential mixed-mode strategy has become standard practice in the survey management of longitudinal studies, in order to achieve consistently high response rates. To realise this aim in a cost-efficient way, a first mode is often an online questionnaire, towards which the target persons are pushed, and a second mode is then a telephone interview, offered to those who do not respond to the initial mode. However, the rationale for using the sequential mixed-mode plus “push-to-web” strategy to reduce the burden of choosing between survey modes, in order to maximise survey participation, could be undermined if there is an overlapping field period during which the target persons could choose between two or more offered modes. The use of reminders might be useful in solving this problem of competing risks. In the context of a multiple-panel study, this question was investigated by utilising longitudinal paradata from the fieldwork, as well as procedures of event history analysis that are adequate for the analysis of processes with competing risks. First, for a web survey as the initial mode and computer-assisted telephone interview (CATI) as the subsequent mode, it was found that the idea of a sequential mixed-mode plus “push-to-web” strategy does work even in the case of competing risks in the choice of a survey mode. Second, it was confirmed that reminders are a useful instrument for stimulating panellists to choose the running survey mode. Third, and finally, it was found that this feature of survey management is effective in countering panellists’ procrastination in regard to responding to a survey.

## Introduction

In recent years, the *sequential mixed-mode strategy* has become standard practice in the survey management of longitudinal studies (Sakshaug et al. 2019: 546; de Leeuw 2018, 2010,2005; Mauz et al. 2018; Klausch et al. 2017; Couper 2017, 2008; Bianchi et al. 2016; Buelens and Brakel 2015; Lynn 2013: 184; Schouten et al. 2013; Millar and Dillman 2011; Börkan 2010; Dillman and Christian 2005; Biemer and Lyberg 2003). In the case of multiple panels in particular, time pressure, extreme high data collection costs in surveys, declining response rates, minimisation of nonresponse bias, and coverage issues, as well as a direct trade-off of mode-specific errors in the total survey error relative to a single mode, are the main reasons for applying this strategy in contrast to *concurrent mixed-mode designs* (Bucks et al. 2020: 353; Sakshaug et al. 2019; Couper 2017; Dillman 2017; Klausch et al. 2017; du Toit 2016; Buelens and Brakel 2015). Reducing sample selection bias is an additional primary motivation for applying sequential mixed-mode methods (Klausch et al. 2017). Meanwhile, some studies show that the sequential mixed-mode method does indeed maximise the response rate, among other potential benefits, and minimises administration costs, which appear to be high for longitudinal surveys (Börkan 2010: 378; Lynn 2013: 184). When seeking to realise these advantages in a multiple panel, the sequence in which different modes are administered can make a difference (de Leeuw 2018; Millar and Dillman 2011). According to Sakshaug et al. (2019: 547), the sequence of a self-administered mode, such as the less expensive and time-consuming computer-assisted web-based interview (CAWI), followed by a more expensive interviewer-administered mode, such as the computer-assisted telephone interview (CATI), results in lower cost compared with the reverse sequence (Greene et al. 2008: 237; Heerwegh 2009: 111; Dillman et al. 2009; Millar and Dillman 2011; Schouten et al. 2013; Buelens and Brakel 2015; Bianchi et al. 2017). Furthermore, starting with a self-administered mode, such as a web survey, instead of an interviewer-administered mode, such as a telephone interview, in a sequential mixed-mode design seems to increase the timeliness and rate of response, as well as improving sample composition, particularly in the context of a longitudinal study (de Leeuw 2005; Manfreda et al. 2006; Groves and Lyberg 2010; Couper 2011; Kreuter 2013; Bianchi et al. 2017; Olson et al. 2020).

To boost response rates by using such a sequential mixed-mode design, in terms of magnitude and timing of survey participation after survey launch, the method of pushing potential respondents to the web mode in sequential mixed-mode surveys (“push-to-web”), followed by a telephone survey as a follow-up mode for nonrespondents of the web phase, seems to be a successful strategy to obtain a maximum of responses by internet before using other modes of response, such as telephone interviews (Dillman 2017). The use of multiple modes of contact through a traditional paper advance letter (with an unconditional monetary incentive enclosed) sent via postage mail and a follow-up email notification (containing the link to the online questionnaire and login information, such as an easily clickable URL and password) is suggested to “push respondents to the web” (Dillman et al. 2009; Millar and Dillman 2011; Dillman 2017; de Leeuw 2018; Becker et al. 2019). This approach allows the invited panellists to access the survey questions immediately and contributes to an improved coverage and response rate by avoiding threats to mode effects in measurement (de Leeuw 2018). The rationale for this approach—i.e. a sequential mixed-mode design with push-to-web procedure—takes into account invitees’ willingness and decision to participate in the survey (Becker et al. 2019; Dillman et al. 2014; Singer 2011; Groves and Couper 1998). On the one hand, the decision on survey participation is facilitated and the cognitive burden of choosing among different modes is avoided (Kleinert et al. 2019; de Leeuw 2018; Lenzner et al. 2009). On the other hand, due to widespread internet use in the target population and the seemingly lower burden and effort for respondents, this is an elaborate and efficient survey mode in multiple-panel studies (Shih and Fan 2008; Couper and Miller 2009; Fan and Yan 2010; Tourangeau et al. 2013; Göritz 2014). For a nonresponse follow-up, CATI offered at a later stage of the field period might be useful for those invitees who have no internet access or who do not prefer this survey mode for several reasons, such as low reading literacy or less computer skills (Millar and Dillman 2011). According to de Leeuw (2010: 1), “mixed-mode surveys try to combine the best of all possible worlds by exploiting the advantages of different modes to compensate for their weakness” (see also: Lynn 2013: 185; Jäckle et al. 2010). The researcher’s decision to mix modes therefore entails an explicit trade-off between errors and the costs of using data collection modes (de Leeuw 2005: 235; du Toit 2016).

However, the rationale for using the sequential mixed-mode design plus push-to-web procedure, in terms of panellists’ decision to participate in a survey, is challenged in a special case that is investigated in this empirical contribution: what happens if target persons are offered a second mode about two weeks after survey launch—due to their nonresponse to the first offered mode—when they are permitted to complete the online questionnaire even in the second stage of the field period? The rationale for this design is to maximise the response rate in the initial survey mode, without any loss of target persons, as well as to minimise time-consuming and costly fieldwork. However, in this case of overlapping access to different modes, problems related to the “paradox of choice” (Schwartz and Ward 2004) between survey modes could arise due to the cognitive overload caused by subjective pressures to decide on one of these options, although this problem should be minimised by sequential offers of different modes (Mauz et al. 2018). The “competing risk” of choosing a survey mode could result in increased nonresponse, or at least in decreasing response rates due to indifference or choice overload. In particular, cognitive overload might be valid for less educated and lower-achieving target persons. Target persons characterised by a low degree of decisiveness are more likely to feel subjective pressures when they have to decide on one of these options. If these assumptions are true, they might explain the mixed results regarding response rates for sequential mixed-mode methods (e.g. Sakshaug et al. 2019: 547; Mauz et al. 2018). In the survey practice of longitudinal studies, reminders are used to minimise this problem. In the first stage, the invited target persons have to decide between (immediate or delayed) participation versus non-participation. Most of them start to complete habitually the online questionnaire in the first three days after receipt (Becker and Glauser 2018; Becker et al. 2019). To stop procrastination in regard to participating in a web survey, up to a maximum of three digital reminders are sent to invitees (e.g. Hoonakker and Carayon 2009). In the second stage, starting almost two weeks after survey launch, the remaining nonrespondents have to decide between three options: use of the initial mode, use of a subsequent mode, or nonresponse. Procrastinating nonrespondents also receive several reminders to push them towards a telephone survey before the field period ends. The question arises whether these additional reminders overcome the respondents’ high cognitive overload due to the “paradox of choice” or their low degree of decision certainty. Since they have experienced the push-to-web procedure in the first stage, the push-to-telephone interview might be helpful for panellists who prefer the second mode, while others could be confused in their decision on survey participation.

Does this sequential offer and its related choice of mode result in different patterns among target persons and a different social composition of survey participation across different stages of the field period? The first research question here is: how many of the panellists, and which of them, take part in one of the two survey modes, and at which point in time during the field period? With respect to nonresponse, does mixing modes result in higher response propensities depending on the mode preferred by different subgroups in the sample, and therefore a more balanced response (less nonresponse bias), as has been assumed by several researchers (e.g. Bianchi et al. 2017)? Furthermore, according to the tailored design method (TDM) suggested by Dillman et al. (2014), invited target persons receive reminders in both stages of the field period to stimulate their survey participation. The second research question is therefore: what role does follow-up reminders sent after the paper prenotification and invitation email play in improving target persons’ willingness to participate in one of the offered survey modes? Are there different effects of reminders on survey participation depending on the survey mode?

Both research questions are investigated in the context of a multiple-panel study on the educational and occupational trajectories of youth born around 1997 and living in German-speaking cantons of Switzerland (Becker et al. 2020a). This panel project has been running since 2012 and eight survey waves have been realised (Becker 2021). A mixed-mode design, including a web survey as the initial mode and a telephone interview as the subsequent mode, has been implemented since the fourth wave, which was conducted in autumn 2014. The empirical analysis focuses on the most recent waves, Waves 7 and 8 (realised in the spring of 2018 and 2020). In particular, the problem of competing risks for survey participation in the second stage of the field period, as well as the role of follow-up reminders, is highlighted by utilising dynamic procedures of event history analysis. Using these statistical procedures, which are adequate for processes with competing risks, it is possible to reveal the subgroup differences in response propensity emerging between modes in the context of a longitudinal survey by controlling for time-constant and time-varying covariates (Blossfeld et al. 2019; Bianchi et al. 2016: 2).

In the remainder of this contribution, in the second section the theoretical background and hypotheses are outlined. The third section describes the data, variables, design, and statistical procedures. The empirical findings are presented in the fourth section. Finally, the fifth section summarises the results and provides a conclusion.

## Theoretical background

### Decision on survey participation and mode preferences

Since participation in a social-scientific panel survey is voluntary, the panellists asked to take part are free to accept or reject that request (Groves and Couper 1998: 1). Even in a sequential mixed-mode design, their decision on survey participation is based on their “free will”. Therefore, they can also choose to take part in their own time (Groves and Couper 1998: 32). They can start completing the questionnaire immediately after the invitation, at a later, more convenient point in time, or never. Thus, survey participation is the result of panellists’ *stochastic decision* (Sigman et al. 2014; Singer 2011). In the context of web surveys, which are often the initial mode of a sequentially mixed-mode design, it is regularly observed that a striking number of experienced panellists participate habitually almost instantly after survey launch. For the other panellists, deliberation about the benefits and costs of online participation is assumed (Singer 2011; Becker and Glauser 2018): their decision takes time, resulting in the time dispersion of their participation (Becker et al. 2019; Green 2014). Therefore, digital reminders (email, SMS) are used to minimise this dispersion by appealing to the nonrespondents to complete the questionnaire.

However, social selectivity of survey participation is often observed across the stages of the fieldwork (e.g. Sakshaug et al. 2019). For example, panellists stemming from higher social classes are more likely to participate (Green 1996: 174). According to Kaminska et al. (2010), survey reluctance—indicated by the time that elapses before the survey recipient responds—is significantly associated with academic ability. The timing of survey participation is thus associated with interviewees’ educational level (Green 1996): that is, a higher level of intelligence and achievement is associated with their earlier response after survey launch. Previous research provides evidence that lower educated persons are less likely to participate in surveys “because they have lower appreciation for this type of research” (Kleinert et al. 2019: 22; Revilla 2012). Therefore, in line with Olson et al. (2012), it is assumed that the panellists’ mode preference, indicated indirectly by their cognitive abilities and social resources, predicts participation in web and phone modes, and the selection of a mode when given the option of two modes. Well-educated and high-achieving target persons are more likely to take part in the survey and to prefer the web mode (*Hypothesis 1.1*). Women often have a higher degree of reading literacy and advanced language proficiency; therefore, they are more likely to take part in a cognitively demanding online survey, while men prefer the telephone mode (*Hypothesis 1.2*).

Since self-administered online questionnaires require advanced cognitive and technological abilities, in terms of reading literacy and computer skills, compared to an interviewer-administered CATI, panellists with a high educational level, advanced language proficiency, and privileged social background are more likely to take part in the initial stage of a survey. For low-achieving panellists, it is useful to offer an interviewer-administered mode to follow up with nonrespondents. For them—the children of working-class parents and of farmers in particular—it might be easier to take part using the subsequent mode, which involves assistance by an interviewer. Therefore, it is assumed that social selectivity of response is much lower for the subsequent mode compared to the initial mode (*Hypothesis 2*).^1^

This means, however, that—provided that there is a “paradox of choice” between survey modes—the mechanisms such as cognitive overload and subjective pressures, caused by subjective pressures to decide on one of these options, are explained by the target persons’ education, academic abilities, and social origin. Furthermore, the respondents’ personality traits such as persistency, control beliefs and decisiveness could explain the outcome of the “paradox of choice”. In this respect, it is assumed that individuals with a low degree of persistency, control beliefs and decisiveness are more likely to choose the subsequent CATI mode—even when the reminders in the second stage of the field period are taken into account (*Hypothesis 3*).

In research on survey methods, it is stressed that spatial and meteorological factors are constraints on survey participation. According to Couper and Groves (1996: 174), large urban areas—inner-city areas in metropolitan areas—generate lower response rates in social-scientific surveys than rural areas (*Hypothesis 4*). Since participating in web-based surveys is primarily an indoor activity (Göritz 2014), panellists are likely start completing online questionnaires in unpleasant weather situations, while telephone interviews—via smartphones in particular – can also be completed as an outdoor activity in “fine” weather. Therefore, it is expected, in the case of respondents’ choice between different survey modes, that participation in CAWI is higher in periods of uncomfortable weather, and the participation in CATI is more likely in periods of comfortable weather (*Hypothesis 5*).

### The role of reminders in the decision on survey participation

The aim of providing multiple reminders is to push as many respondents as possible to complete the survey option, targeting those who are unwilling to respond to the first offered mode (Millar and Dillman 2011). However, the effect of multiple reminders on response rates has not been thoroughly investigated in longitudinal studies, or in sequential mixed-mode designs (Christensen et al. 2014). Therefore, the effects of multiple personalised reminders on response rates are additionally examined in the context of a multiple-panel study (Muñoz-Leiva et al. 2010). According to Dillman et al. (2014), research on survey methods has found that multiple contacts directed at target persons after survey launch via reminders and mixed-delivery strategies (email or SMS) have a positive effect on the response rates (Schaefer and Dillman 1998; Dillman 2000; Klofstad et al. 2008; Rao and Pennington 2013; Christensen et al. 2014; Van Mol 2017; Becker and Glauser 2018; Langenderfer-Magruder and Wilke 2019). Hoonakker and Carayon (2009) recommend sending at least three reminders at three-day intervals after sending out the advance letter of invitation, since web-based surveys show that most responses occur within the first three days. Therefore, it is assumed that follow-up contacts generally are a powerful technique for increasing response rates across subsequent web and telephone modes (*Hypothesis 6*).

In line with the experimental studies by Crawford et al. (2001) and Deutskens et al. (2004), it has been found that early reminders are more effective in each of the considered modes, and provide stronger effects on panellists’ participation (Muñoz-Leiva et al. 2010). Additionally, Van Mol (2017) found that extra reminders are helpful in raising response rates among populations that are “over-surveyed”. This could be true for experienced panellists, providing for panel fatigue in the most recent panel waves. At the end of the field period, nonrespondents who postpone their decision on survey participation are more likely to ignore even these extra reminders. Therefore, it is expected that the effect of reminders decreases the longer the nonrespondents remain undecided about their participation and choice of a survey mode (*Hypothesis 7*).

However, does a reminder sent at the second stage of the field period after offering the subsequent telephone mode to the remaining nonrespondents only increase responses to the second mode, or does it also increase responses to the initial online mode? Since the reminders are related directly to the mode currently offered, it is assumed that reminders offered at the second stage do not increase the participation in the online survey, but only in the CATI mode (*Hypothesis 8*).

## Data, design, variables, and statistical procedures

### Data set and design

The empirical analysis is based on longitudinal data from the DAB panel study (Becker et al. 2020a). The aim of the panel study is the mechanism-based investigation of the educational and occupational trajectories of youth born around 1997 and living in German-speaking cantons of Switzerland. The sample of this target population is random. The target population of the DAB study consists of eight-graders of the 2011/12 school year who were enrolled in regular classes in public schools. The panel data are based on a random and 10% stratified gross sample of 296 school classes, out of a total universe of 3045 classes. A disproportional sampling of school classes from different school types, as well as a proportional sampling of school classes regarding the share of migrants within schools, was applied. At school level, a simple random sample of school classes was chosen. The initial probability sampling is based on data obtained from the Swiss Federal Statistical Office (Glauser 2015).

The panel study started in 2012. In the first three surveys, the target persons were interviewed in the context of their school class via online questionnaire. After that, they left compulsory school and have had to be pursued individually, since summer 2013. Therefore, a sequential mixed-mode design was established, including the TDM suggested by Dillman et al. (2014). Since the fourth wave, the eligible panellists have been pushed towards the web-based online mode by a personalised advanced invitation letter, including an incentive, sent by regular postal mail (Becker et al. 2019). Using the fast option offered by Swiss Post, the A-post, it is guaranteed that eligible target persons will receive this letter the next day. They are informed that the panel study is financed by the Swiss Secretary of Education, Research and Innovation (SERI), a governmental agency, and that it is conducted by a team of researchers at a cantonal university. One day later, they receive the clickable URL and password to log on to the web site by email. If they do not start to complete the questionnaire after some days, they get personalised reminders. About two weeks after survey launch, nonrespondents are invited to take part in CATI. If they do not react to call attempts and reminders, a traditional paper-and-pencil survey is offered as a final mode.

Eight surveys have been realised. For the current research issue, analyses are focused on the field period of the two most recent waves, conducted in May/June 2018 and 2020. In both waves, the eligible panellists received an unconditionally prepaid monetary incentive enclosed in the invitation letter (Becker et al. 2020a). To test the hypotheses, paradata from the first mode—the web-based online survey—are used, providing accurate time references of field periods and individuals’ survey participation. In the online mode, the personalised reminders were sent about four, seven, and 10 days after the survey launch (i.e. at three-day intervals). The first reminder was a text sent by SMS; the second was an SMS or email; and the third was an email. After about 12 days, the nonrespondents were informed about a contact for the CATI. For each contact the exact time and status references were documented. After three call attempts the nonrespondents got a reminder via SMS. Three weeks after survey launch, they got an email reminding them to take part in the CATI. The total field period lasted 40 days for the seventh wave and 52 days for the eighth wave.

### Dependent and independent variables

There are two dependent variables. The first is the *respondents’ likelihood of taking part in the survey* at any point during the field period. This distinguishes between participation in the online and CATI modes. The second variable is the *respondents’ likelihood of receiving an (electronic) reminder* during the field period across both survey modes.

For the independent variables, different analytical levels are considered. At the *macro level*, the *weather situation* and the *regional opportunity structure* are taken into account. The weather situation is measured using time series delivered by the Federal Office of Meteorology and Climatology (2020) on a daily basis, considering weather characteristics during the field periods, such as average air temperature by day (in degrees centigrade), relative humidity (daily average percentage), rainfall (daily average in millimetres), duration of sunshine (in hours per day), and barometric pressure (in hectopascals), and extracted by confirmatory factor analysis (Becker 2021). The opportunity structure of the region in which the panellists live is measured by macro data from the Swiss Federal Statistical Office and reflects the principle of small, partially cross-cantonal labour market areas with functional orientation towards centred and peripheral opportunities and living standards, in addition to urbanicity, population density, and lack of social cohesion (Glauser and Becker 2016: 20).

At the *meso level* of survey characteristics, the *number of panel waves* is considered as a dummy variable. This is also true for the *number and different types of reminders* delivered to the target persons.

At the *micro level* of target units, first of all, their *social origin* is indicated by their parents’ social class. This is measured by the class scheme suggested by Erikson and Goldthorpe (1992). Second, their *enrolment in secondary school* until the end of compulsory schooling is taken into account. This distinguishes between different educational levels, considering requirements such as basic, extended, and advanced levels relevant for the ability to read and complete a questionnaire. Furthermore, the panellists’ *gender*, as well as their *language proficiency* measured by the standardised grade average points in German, correlating with their reading literacy, is taken into account. Finally, panellists’ personality traits, such as persistence, control beliefs, and decisiveness, are taken into account, since these characteristics are seen as significant for willingness to take part in a social-scientific survey (Saßenroth 2013), as well as for the choice of the survey mode offered to them.^2^

### Statistical procedures

Since participation in push-to-web surveys or CATI is modelled as a *time-dependent stochastic process* of an individual’s decision on participation and selection of survey mode, which could occur at each of the points in time across the field period, *event history analysis* is applied to reveal causal endogenous and exogenous factors influencing the likelihood and timing of survey participation, as well as the effect of reminders on survey participation (Tourangeau et al. 2013: 38). By considering time-varying covariates in an event-oriented design, it is possible to reveal the causalities of this stochastic process (Rohwer and Blossfeld 1997; Pötter and Blossfeld 2001). In regard to statistical analysis, event history analysis provides techniques and procedures to take these theoretical and methodological premises into account (Blossfeld et al. 2019: 1–40).

For the longitudinal analysis, different procedures of *event history analysis* are utilised to analyse the time until interesting events—such as survey participation, selection of one of the survey modes, or receiving a reminder—occur within the field period. However, due to the *sequential mixed-mode design*, specialities of the timing of events have to be considered. In the sequential mixed-mode design, access to the online mode is possible for each of the invitees during the complete field period. As mentioned above, the nonrespondents among them are asked, about two weeks after survey launch, to take part in the CATI mode. This means there is then a competing risk of taking part in one of the two offered modes, which are mutually exclusive during an overlapping risk period. A competing risk is an event—such as participation in one of the two survey modes—that either hinders the occurrence of the primary event of interest (e.g. participation in the online survey instead of CATI) or that modifies the chance that this event (e.g. participation in CATI) occurs (Noordzij et al. 2013: 2670). For example, when analysing participation in the online survey (initial mode) towards which potential respondents are pushed at survey launch, inviting nonrespondents to take part in the CATI (subsequent mode) about two weeks after the survey launch is an event that competes with the acceptance of the initial mode as the primary event of research interest. When an eligible panellist chooses one mode or another, the unchosen mode cannot be realised at another point in time, due to censoring. However, panellists who have not started completing the online questionnaire have the “*chance*” to take part in the CATI or online mode at a point in time that is convenient for them. According to Schwartz (2009), due to the burden of an additional option, they could be spoilt for choice. In this case, it is likely that they will not take part in the survey. Another positive outcome is that the individual’s preference for CATI hinders them in starting the online questionnaire. Unintended by the researchers, it could occur that offering the CATI mode pushes nonrespondents towards the online mode. Finally, it could be likely that survey participation will be postponed due to indifference or choice overload.

According to Schuster et al. (2020), estimations could be biased systematically when competing events—i.e. two or more cause-specific hazards (Kalbfleisch and Prentice 2002)—are ignored in the analysis of survival data. Against the background of competing risk—the potentially simultaneous occurrence of mutually exclusive events, such as participation in the online mode versus the CATI mode, in overlapping stages of the field period—the traditional survival analysis (i.e. Kaplan–Meier product-limit estimations) is inadequate for describing the timing and likelihood of panellists’ survey participation. The assumption of standard survival analysis, namely that the censoring of events is independent, is not valid in this case. Therefore, the Kaplan–Meier estimator is biased since the probability of the event of primary interest is overestimated (Noordzij et al. 2013: 2672). The overestimation of probabilities increases with risk time. Therefore, alternative *nonparametric procedures* of competing risk analysis—the *cumulative incidence competing risk method*—are used to describe the panellists’ participation patterns across the field period. Since Kaplan–Meier plots are biased in the presence of competing risks, the *cause-specific cumulative incidence function* (CIF), which is the probability of survey participation before the end of field period $t$, is estimated to reveal the risk of choosing one of the competing survey modes (Lambert 2017). The CIF describes the incidence of the occurrence of an event while taking competing risks into account (Austin and Fine 2017: 4293).

In particular, the *piecewise constant analysis* is also used to describe the hazard rates for receiving reminders, as well as panellists’ survey participation, to reveal the effect of reminders on panellists’ reactions at each point in time during the field periods. According to Blossfeld et al. (2019: 124), the basic idea of this procedure is to split the time axis into time periods (e.g. on a daily basis) and to assume that transition rates are constant in each of these intervals but can change between them. Using this procedure, it is possible to describe the occurrence of (competing) events in different phases of the field period. Given theoretically defined time periods, the transition rate for survey participation is defined as follows: $$r_{k} \left( t \right) = exp\left\{ {\overline{\alpha }\begin{array}{*{20}c} {\left( k \right)} \\ I \\ \end{array} + A^{\left( k \right)} \alpha^{\left( k \right)} } \right\} if t \in I_{t}$$, whereby k is the destination, *I* the time interval, $$\overline{\alpha }\begin{array}{*{20}c} {\left( k \right)} \\ I \\ \end{array}$$ is a constant coefficient associated with the *lth* time period, $A^{\left( k \right)}$ is a vector of covariates, and $\alpha^{\left( k \right)}$ is an associated vector of coefficients assumed not to vary across time (Blossfeld et al. 2019: 125). This model is estimated without any covariates, since crude hazard rates should be estimated across the time interval of the field periods.

Furthermore, *parametric regression procedures* are used to estimate the impact of independent variables on the likelihood of interesting events. The hazard rate $r\left( t \right)$ is defined as the marginal value of the conditional probability of such an event occurring—namely the instantaneous rate for survey participation or receiving a reminder—in the time interval $\left( {t, t + \Delta t} \right)$, given that this event has not occurred before time $t$ (Blossfeld et al. 2019: 29). First of all, for single events, such as survey participation, or repeated events, such as receiving reminders across the field period, the hazard rate is estimated on the basis of an *exponential model*:$r\left( {t|x\left( t \right)} \right) = \exp \left( {\beta^{\prime } x\left( t \right)} \right)$, whereby $x\left( t \right)$ is the time-dependent vector of exogenous variables whose unknown coefficients *β* have to be estimated. To account for time-varying covariates, the technique of *episode splitting* is used: i.e. the initial waiting time is split into sub-episodes on a daily basis. For each of these sub-episodes, a constant hazard rate is assumed. By applying this procedure, it is possible to model step functions displaying the empirically observed hazard function for the entire process until participation or getting a reminder.

In the case of competing risks in terms of participation in the online survey versus the CATI, the *exponential model (including the episode splitting)* is equivalent to the *proportional cause-specific hazards model* suggested by Kalbfleisch and Prentice (2002). According to Schuster et al. (2020: 44), the “cause-specific hazard denotes the instantaneous rate of occurrence of the event of interest in a setting in which subjects can also experience the competing event”. Since this hazard is estimated by removing individuals from the risk set the moment they experience the competing event, meaning that competing events are treated as censored observations, it is possible to estimate the cause-specific hazard using an exponential model in which all events other than the event of interest are treated as censoring. Schuster et al. (2020: 44) suggest interpreting these hazard ratios “among subjects who did not (yet) experience the event of interest or a competing event. As the cause-specific hazard is directly quantified among subjects that are actually at risk of developing the event of interest, the cause-specific hazard model is considered more appropriate for etiological research.” This is realised by calculating estimations of survey participation separately for the different modes. However, according to Lunn and McNeil (1995: 524), these methods provide the drawback “that [they do] not treat the different types of failures jointly, complicating the comparison of parameter estimates corresponding to different failure types”.

Another approach—the *subdistribution hazards approach* by Fine and Gray (1999)—is often seen as the most appropriate method to use for analysing competing risks. In contrast to the cause-specific hazards model, “subjects who experience a competing event remain in the risk set (instead of being censored), although they are in fact no longer at risk of the event of interest” (Noordzij et al. 2013: 2673). This precondition is necessary to establish the direct link between the covariates with the CIF to predict the hazard ratios. However, this makes it difficult to interpret them in a straightforward way, and is therefore not appropriate for etiological research (Schuster et al. 2020: 44). By taking competing risks into account, the coefficients estimated by the *stcrreg* module implemented in the statistical package *Stata* can be used to compute the cumulative incidence of participation in one of the survey modes, and to depict the hazards in a CIF plot. In sum, the “cause-specific hazard model estimates the effect of covariates on the cause-specific hazard functions, while the Fine-Gray subdistribution hazard model estimates the effect of covariates on the subdistribution hazard function” (Austin and Fine 2017: 4393).

## Empirical results

### Description of response pattern in sequential mixed-mode design

The overall response rate in Wave 7 was about 76% and in Wave 8 it was about 81%. In each of the waves, the response rate was higher for women than for men (Wave 7: 78% versus 74%; Wave 8: 84% versus 78%). The share of dropouts was less than 1‰ overall.

**Fig. 1:**
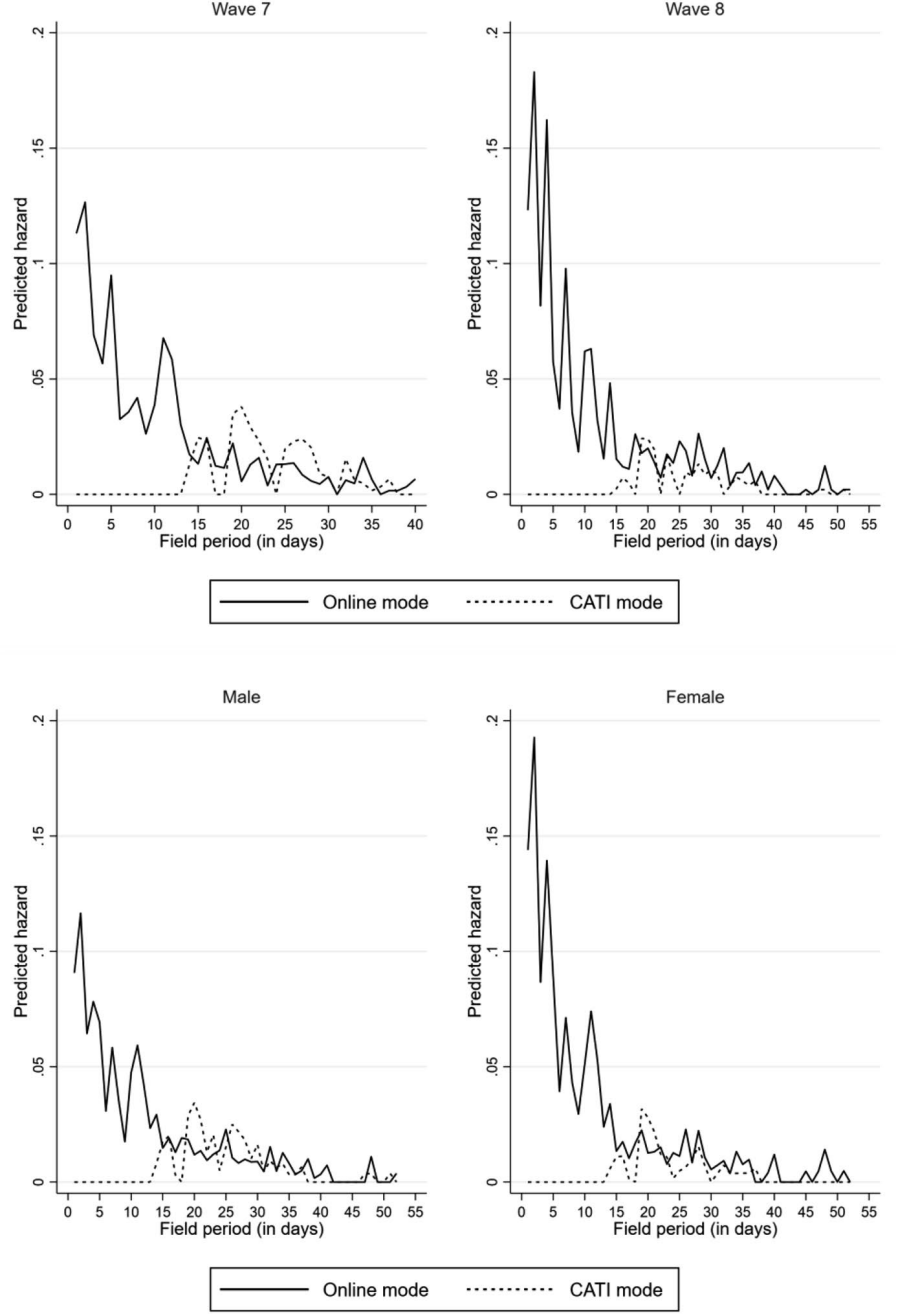
Comparison of the hazard rates of different survey modes—Piecewise constant exponential model

Due to the statistical problems of survival analysis in the case of competing risks, the crude hazard rates are estimated by applying a piecewise constant rate model to describe the time-dependent pattern of participation at different points in time and for different survey modes. The predicted hazard rate refers to the rate of survey participation for panellists at risk of a given time in the field period. It defines the likelihood per time interval of a day that nonrespondents will take part in the survey in this time interval. The graphs in the upper panel of Fig. 1 depict higher hazard rates in Wave 8 for participation in the online mode compared to Wave 7; conversely, the hazards for the CATI mode are much higher in Wave 7. In the lower panel, it becomes obvious that hazard rates in the initial mode are lower for men than for women. For the subsequent mode, a reverse pattern is observed for the genders. The lines of hazard rates across field periods are jagged. It could be the case that the convexities indicate effects of reminders on panellists’ likelihood of taking part in one of the offered survey modes.

**Fig. 2:**
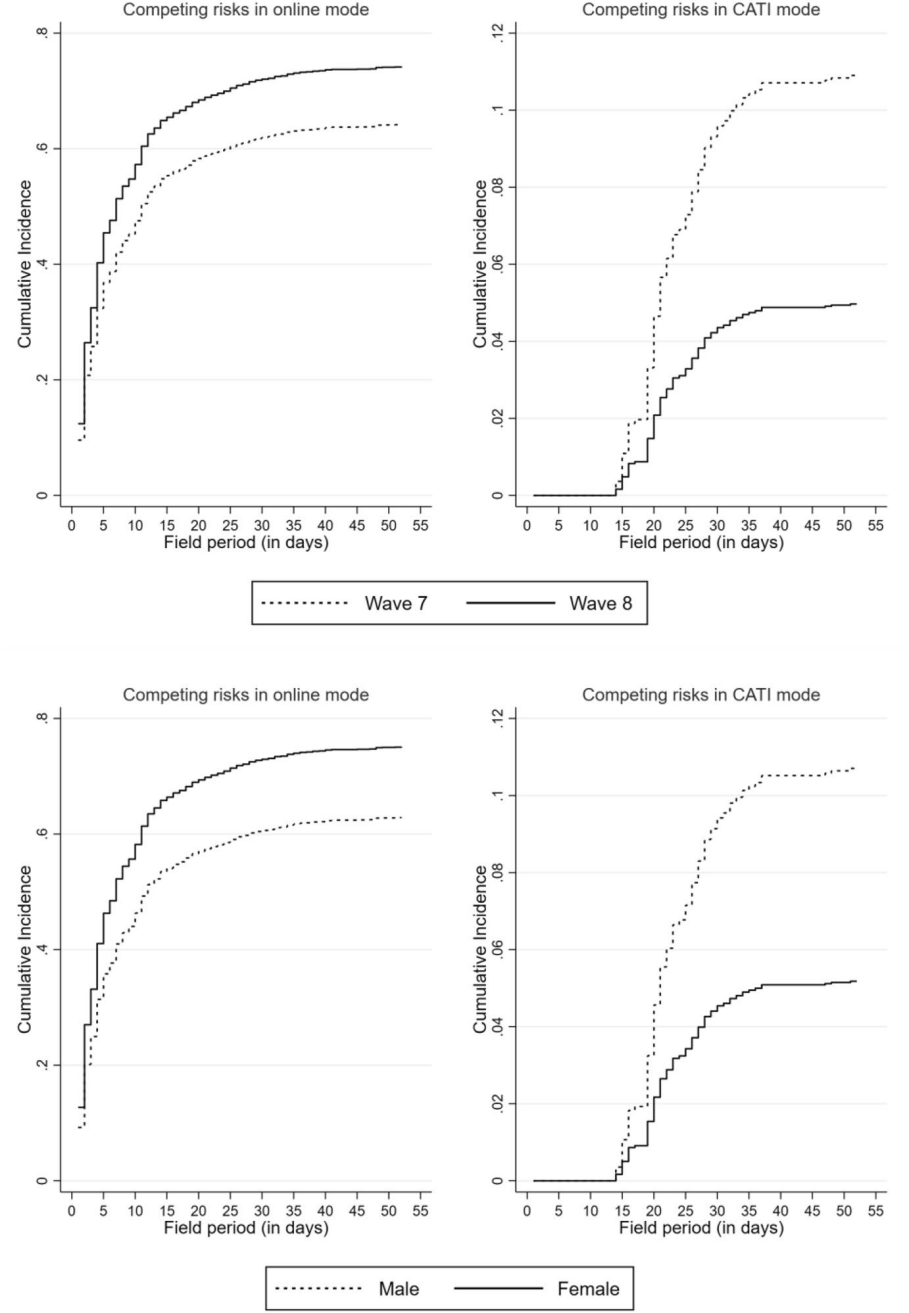
Competing risk for survey participation by waves and gender—CIF (episode splitting)

The differences in survey participation patterns are more evident when the hazards are depicted as cumulative incidences in a CIF plot. In the upper panel of Fig. 2, the wave differences are shown separately for both survey modes. On the one hand, it is confirmed that the likelihood of participating in the online survey was higher in Wave 8 than in Wave 7, where there was stagnation after a month.^3^ Across the waves, the role of the web survey became more significant for the eligible panellists.

On the other hand, the likelihood of participating in the CATI mode increased in Wave 7 much faster than in the subsequent wave. In both waves, the likelihood of participating in the CATI mode stagnated after three weeks. These patterns are similar for the genders depicted in the lower panel. While women were more likely to take part in the initial mode, men preferred to take part in the CATI mode.

In sum, however, the hazards are significantly lower for the subsequent mode. This mode difference between women and men could contribute to explaining gender-specific differences in survey participation, whereby participation rates are higher for women compared to men (Green 1996).

### Survey participation and choice of survey modes

For the multivariate test of hypotheses on survey participation, we distinguish between two approaches: the cause-specific and the subdistribution hazard models. Considering both survey modes, the competing risk behind the panellists’ decision on their survey participation is taken into account by separate estimations for participation in the online survey (CAWI) and participation in the telephone survey (Table 1).

**Table 1:** Survey participation and competing risk between survey modes

Approaches/models	Cause-specific hazard models		Subdistribution hazards approach	
	1.1^1^: CAWI	1.2^1^: CATI	2.1^2^: CAWI	2.2^2^: CATI
*Macro factors†*				
Weather situation	− 0.258	0.698	− 0.032	0.569
	(0.020)***	(0.047)***	(0.026)	(0.109)***
Regional opportunity structure	− 0.070	− 0.028	− 0.053	− 0.021
	(0.024)**	(0.058)	(0.017)**	(0.056)
*Waves (Ref.: Wave 7)†*				
Wave 8	0.198	− 0.785	0.254	− 0.744
	(0.045)***	(0.106)***	(0.033)***	(0.115)***
*Social origin (Ref.: missing value)*				
Upper service class	0.290	0.217	0.218	0.171
	(0.089)**	(0.194)	(0.063)***	(0.188)
Lower service class	0.238	0.297	0.185	0.254
	(0.084)**	(0.179)	(0.060)**	(0.174)
Routine non-manual employees	0.233	0.235	0.164	0.158
	(0.079)**	(0.173)	(0.057)**	(0.168)
Farmers, small proprietors	0.241	0.732	0.149	0.696
	(0.114)*	(0.222)***	(0.080)	(0.213)**
Foremen, skilled manual workers	0.078	− 0.110	0.068	− 0.150
	(0.085)	(0.191)	(0.063)	(0.186)
Semi/unskilled manual workers	0.188	0.528	0.092	0.451
	(0.111)	(0.239)*	(0.082)	(0.233)
*School type (Ref.: miscellaneous type)*				
Basic requirements	− 0.411	− 0.232	− 0.314	− 0.197
	(0.085)***	(0.175)	(0.064)***	(0.171)
Extended requirements	0.225	− 0.066	0.154	− 0.085
	(0.078)**	(0.164)	(0.056)**	(0.160)
Advanced requirements	0.850	0.330	0.559	0.177
	(0.086)***	(0.218)	(0.060)***	(0.213)
*Other individual characteristics*				
Female	0.283	− 0.487	0.248	− 0.472
	(0.046)***	(0.111)***	(0.033)***	(0.108)***
Language proficiency	0.159	− 0.023	0.121	− 0.030
	(0.026)***	(0.055)	(0.019)***	(0.054)
Persistence	0.056	− 0.033	0.041	− 0.048
	(0.026)*	(0.057)	(0.019)*	(0.056)
Control belief	0.046	0.032	0.029	0.030
	(0.025)	(0.056)	(0.018)	(0.054)
Decisiveness	0.063	–0.052	0.055	− 0.046
	(0.027)*	(0.054)	(0.020)**	(0.052)
*Reminders†*				
Online mode: Reminder #1	1.209		0.769	
	(0.050)***		(0.085)***	
Online mode: Reminder #2	0.518		0.629	
	(0.073)***		(0.124)***	
Online mode: Reminder #3	0.483		0.263	
	(0.082)***		(0.120)*	
CATI: First reminder (SMS)		1.707		1.045
		(0.173)***		(0.170)***
CATI: Follow-up reminders (SMS)		0.791		0.828
		(0.166)***		(0.185)***
CATI mode: Reminder (Email)		0.720		0.134
		(0.194)***		(0.166)
*Constant*	− 3.967	− 4.521		
	(0.082)***	(0.174)***		
Number of episodes / cases	82,888 / 4,986	43,854 / 1,985	82,888 / 4986	43,854 / 1985
Number of events	3,495	417	3,495	417
Number of censored / competing cases	1074 / 417	174 /373	1074 / 417	1074 /373
Wald chi^2^ (d.f.)	1607.04 (20)	706.59 (20)	787.42 (20)	182.54 (20)

^*^ *p* < 0.05; ** *p* < 0.01; *** *p* < 0.001; β-coefficients, estimated by ^1^exponential model and ^2^competing risk model (with episode splitting in brackets: standard error); † time-varying covariates

Considering, first, the time-varying macro and meso factors, it is found that a pleasant weather situation had negative impacts on the likelihood of completing the online questionnaire (Models 1.1 and 2.1), while taking part in the CATI was more likely in “fine” weather situations (Models 1.2 and 2.2). Thus, *Hypothesis 5* is confirmed empirically.

In line with *Hypothesis 4*, the likelihood of participating in CAWI was significant lower in urban areas than in rural areas (Models 1.1 and 2.1). Since regional opportunity structures had no significant impacts on CATI participation (Models 1.2 and 2.2), it could be assumed that the high urbanicity and living standard of the regional context, providing tempting prospects for outdoor activities, diverted the panellists living there from starting an indoor activity such as an online questionnaire. In contrast, telephone access is universal and CATI can be conducted as an indoor or an outdoor activity all day long. The previous graphical findings are confirmed by the multivariate estimations. Panellists were more likely to start with the completion of the online questionnaire in Wave 8, while participation in CATI was more likely to take place in Wave 7.

In addition to these heterogeneities, there was a different social selectivity in survey participation for the survey modes considered in this panel study. First of all, *Hypothesis 1.1* is confirmed, emphasising the correlation between panellists’ mode preference, abilities, and social resources. Second, in contrast to the choice of online questionnaire, language proficiency and related achievements in the German language (such as reading literacy and cognitive abilities) had no significant impact on the choice of CATI. As a theoretically unintended by-product, it is revealed again that women preferred participation in the self-administered mode, in contrast to men, who were more likely to use the interviewer-administered telephone mode. This is in line with *Hypothesis 1.2*.

Panellists from the upper and middle social classes who were enrolled in intermediate and upper secondary school types and who provided their favourite language proficiencies were more likely to take part in the online survey. The social selectivity in terms of social origin was much lower for CATI choice and participation. Children of farmers, small proprietors, and semi-skilled and unskilled manual workers preferred participating in the telephone mode. These results confirm *Hypothesis 2* on significant lower selectivity for the subsequent mode. They indicate that there is no “paradox of choice” in terms of the participation in one of the two offered modes. The amount, timing and selectivity of survey participation across the field period depended on individual resources and abilities.

Furthermore, controlling for other covariates, estimations show that personal traits played a minor role in survey participation. As might be expected, panellists demonstrating a high degree of decisiveness and persistence at the start were more likely to complete the online questionnaire (Models 1.1 and 2.1). However, the effects were low compared to other impacts. For the choice of CATI, there was no effect of such personal characteristics. This result is in contradiction to *Hypothesis 3*. It indicates again, indirectly at least, that the thread of negative consequences due to “paradox of choice” between the survey modes is overrated. This might be true when the effects of reminders are considered, which could be useful for the dissolution of cognitive overload among procrastinating respondents.

Finally, the impact of reminders on survey participation and mode choice is investigated. The estimations are straightforward. In line with *Hypothesis 6*, emphasising that follow-up contacts are a powerful technique for increasing response rates across subsequent survey modes, it is found, by applying the cause-specific hazard models, that each of the reminders—independent of the number and type of reminders—strengthened the panellists’ propensity for survey participation. The subdistribution hazards approach, however, underestimated the impact of the last reminders in the online and telephone mode. Indeed, the impact of reminders decreased as their number increased, with the first reminders sent in the early stages of the field period more likely to trigger the nonrespondents to take part in the survey. In line with *Hypothesis 7*, it is found that, at the end of the field period, the interviewees who were still at risk for response were more likely to ignore even these extra reminders.

**Fig. 3:**
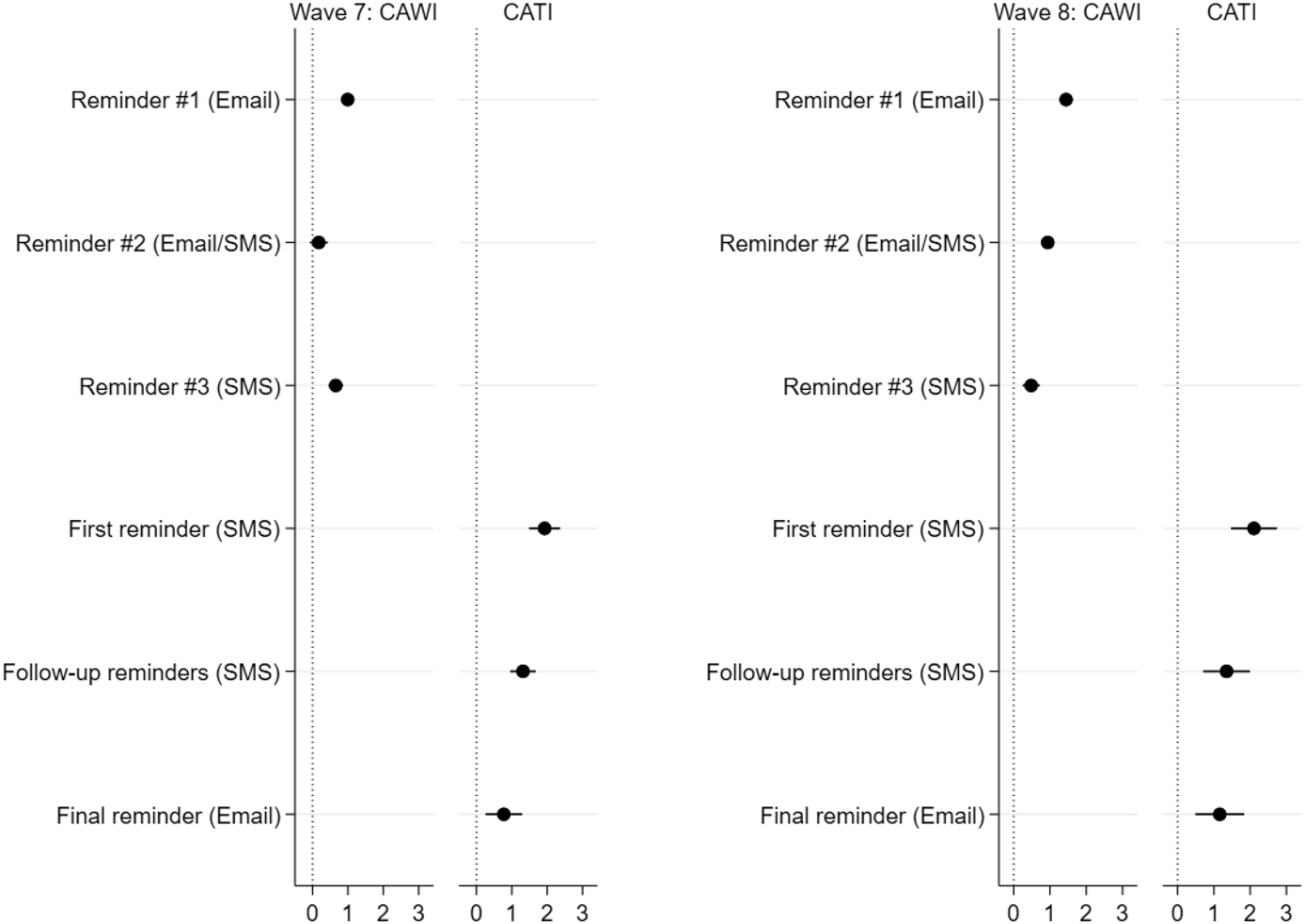
Competing risk for survey participation—Effect of reminders (β-coefficients)

In Fig. 3, it is documented that this conclusion is true for each of the waves and different survey modes. For each of the survey modes, it is true that the first reminders sent in an early period of the fieldwork provided the greatest effects intended by the researchers. The follow-up reminders showed lower effects on participation compared to the initial reminders.

**Fig. 4:**
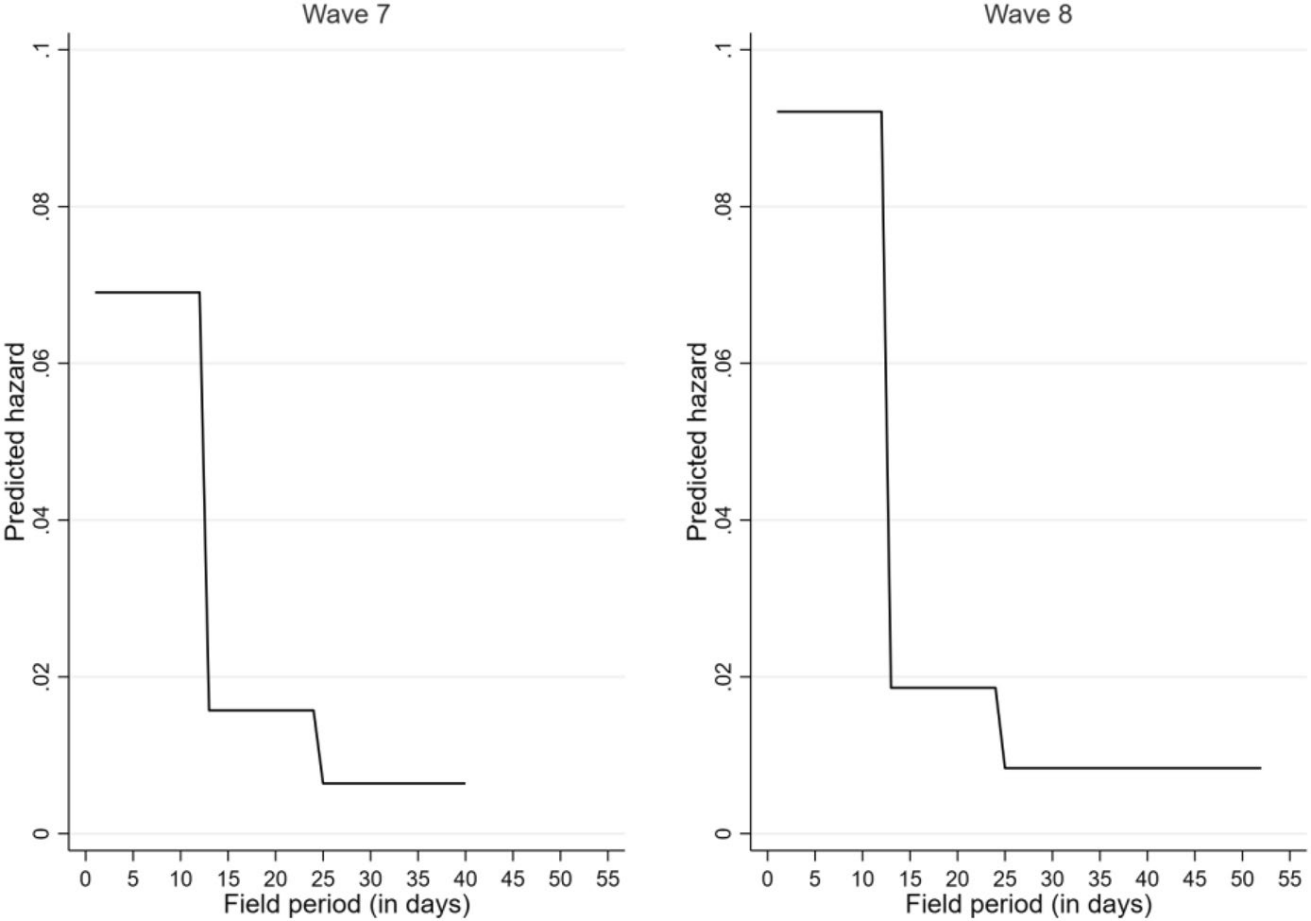
Impact of CATI notification on participation in the online survey across waves

In a next step, it is confirmed that there is a correlation between the timing of reminders and the panellists’ choice of mode. First of all, in Fig. 4, it becomes obvious for Waves 7 and 8 that a CATI notification indicating the launch of the subsequent CATI mode did not result in an increasing participation in the initial online mode.

**Fig. 5:**
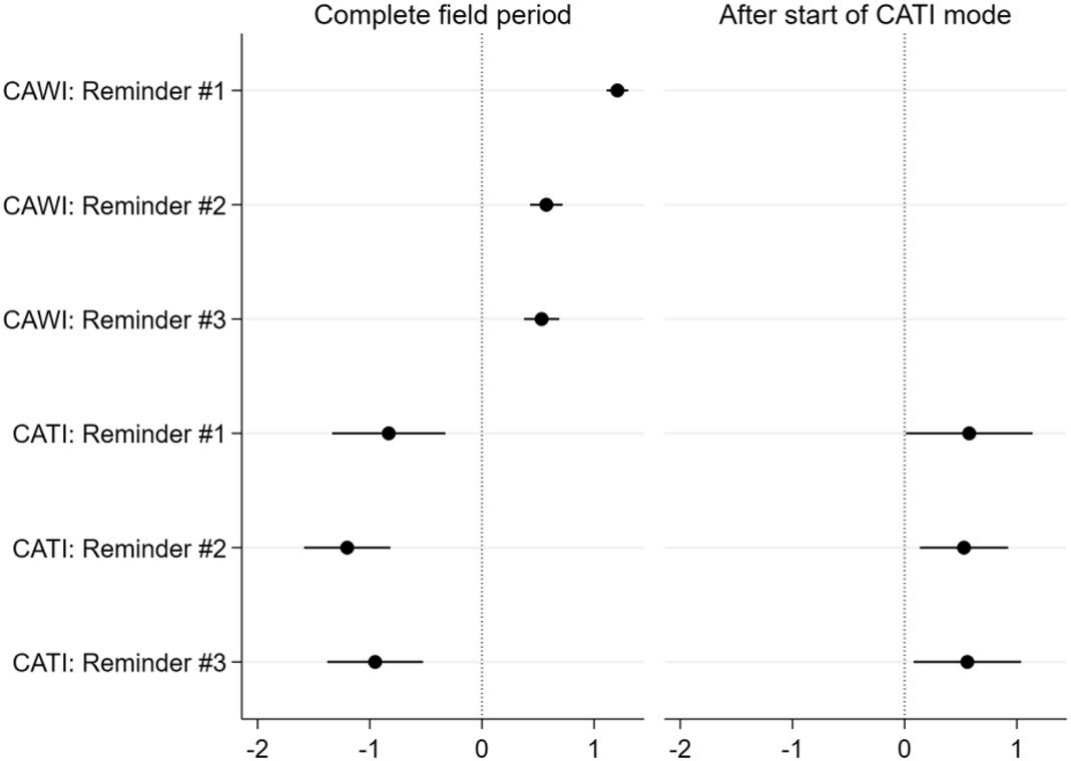
Impact of different reminders on participation in the online survey—Cause-specific hazard model (β-coefficients)

Second, additional estimations—depicted in Fig. 5—provide results confirming *Hypothesis 8*. Indeed, reminders offered at the stage after the request to participate in the subsequent mode did not increase participation in the initially offered online survey; rather, they increased participation in the CATI mode. If one considers the complete field period, it becomes evident that only reminders offered for the first time after survey launch—i.e. at the stage when access to the online questionnaire was exclusive—contributed to increased participation in the web-based survey. Reminders offered during the time interval when access to both the online survey and to CATI was simultaneously possible resulted in a decreasing likelihood of participating in the initial online mode.

Third, if only the nonrespondents at the launch of the subsequent mode are considered, it is found that the reminders encouraged them to complete the online questionnaire. This finding again emphasises the advantage of a sequential mixed-mode design.

### Who gets reminders in the initial and subsequent survey mode?

In the final step of empirical analysis, the following question should be answered: who needs, and who gets, these reminders? To the best of our knowledge, this question has not been answering by applying dynamic multilevel estimations in a longitudinal design before. The estimations provide interesting results that are instructive for the practice of survey management (Table 2).

**Table 2:** Likelihood of reminders in the CAWI and CATI modes

Models/	1.1	1.2	1.3	2.1	2.2	2.3
reminders	CAWI #1	CAWI #2	CAWI #3	CATI #1	CATI #2	CATI #3
*Macro factors*						
Weather situation	0.779	− 0.286	− 0.284	1.082	1.731	4.484
	(0.013)***	(0.003)***	(0.041)***	(0.024)***	(0.045)***	(0.113)***
Regional opportunity structure	0.005	0.005	0.018	0.073	0.051	− 0.002
	(0.006)	(0.008)	(0.009)*	(0.037)*	(0.035)	(0.006)
*Waves (Ref.: Wave 7)*						
Wave 8	0.659	− 0.094	0.002	− 0.785	− 0.702	− 3.777
	(0.012)***	(0.014)***	(0.018)	(0.070)***	(0.070)***	(0.109)***
*Social origin (Ref.: missing value)*						
Upper service class	− 0.107	− 0.076	− 0.079	0.025	0.081	− 0.028
	(0.024)***	(0.028)**	(0.034)*	(0.138)	(0.125)	(0.019)
Lower service class	− 0.070	− 0.086	− 0.075	0.166	0.025	− 0.005
	(0.021)***	(0.026)***	(0.031)*	(0.122)	(0.116)	(0.016)
Routine non-manual employees	− 0.054	− 0.058	− 0.048	0.116	− 0.050	− 0.025
	(0.019)**	(0.023)*	(0.027)	(0.116)	(0.110)	(0.016)
Farmers, small proprietors	− 0.027	− 0.112	− 0.088	0.147	− 0.020	− 0.027
	(0.027)	(0.039)**	(0.044)*	(0.176)	(0.170)	(0.026)
Foremen, skilled manual workers	− 0.042	− 0.036	0.002	− 0.126	− 0.151	− 0.001
	(0.020)*	(0.024)	(0.027)	(0.124)	(0.116)	(0.015)
Semi/unskilled manual workers	− 0.011	− 0.044	− 0.022	0.261	− 0.041	− 0.023
	(0.026)	(0.034)	(0.038)	(0.163)	(0.167)	(0.024)
*School type (Ref.: other types)*						
Basic requirements	0.054	0.054	0.068	− 0.179	0.052	0.004
	(0.021)*	(0.025)*	(0.028)*	(0.121)	(0.116)	(0.016)
Extended requirements	− 0.012	− 0.058	− 0.081	− 0.085	0.027	− 0.013
	(0.021)	(0.025)*	(0.029)**	(0.118)	(0.114)	(0.016)
Advanced requirements	− 0.158	− 0.246	− 0.306	− 0.026	0.090	− 0.043
	(0.029)***	(0.037)***	(0.045)***	(0.161)	(0.160)	(0.027)
*Other individual characteristics*						
Female	− 0.083	− 0.088	− 0.098	− 0.148	− 0.050	0.005
	(0.012)***	(0.015)***	(0.018)***	(0.073)*	(0.069)	(0.010)
Language proficiency	− 0.037	− 0.034	− 0.036	− 0.021	0.008	− 0.006
	(0.007)***	(0.009)***	(0.010)***	(0.042)	(0.039)	(0.005)
Persistence	− 0.013	− 0.012	− 0.009	− 0.039	− 0.044	− 0.009
	(0.006)*	(0.008)	(0.009)	(0.037)	(0.036)	(0.005)
Control belief	− 0.003	− 0.011	0.002	0.005	0.021	− 0.001
	(0.006)	(0.008)	(0.009)	(0.038)	(0.036)	(0.005)
Decisiveness	− 0.009	− 0.024	− 0.023	− 0.040	− 0.013	0.002
	(0.007)	(0.008)**	(0.009)*	(0.040)	(0.038)	(0.005)
*Constant*	− 1.527	− 2.353	− 2.765	− 2.740	− 3.454	0.736
	(0.020)***	(0.023)***	(0.041)***	(0.121)***	(0.113)***	(0.061)***
Number of episodes	18,234	26,602	33,869	26,479	31,441	14,256
Number of individuals	4986	4986	4986	1985	1985	185
Number of events	3336	2679	2349	1058	813	1574
Wald chi^2^ (d.f.)	6202 (17)	11,274 (17)	364 (17)	2001 (17)	1644 (17)	5277 (17)

^*^p < 0.05; ** *p* < 0.01; *** *p* < 0.001; β-coefficients, estimated by exponential model (with episode splitting in brackets: standard error)

Controlling for external circumstances, such as weather situation, regional opportunity structure and panel waves, it is plausible—if the timing and number of reminders is considered—that panellists from upper and intermediate social classes were less likely to get a reminder, in contrast to their counterparts, since they were more likely to respond to the request for the online survey, as revealed above (Models 1.1–1.3). In line with the previous finding for participation in the CATI mode, there is no selectivity in terms of the panellists’ social origin (Models 2.1–2.3).

Regarding panellists’ educational level, there is also a reflection of the pattern of survey participation reported in the previous part, above. Less educated panellists were more likely to get reminders in the initial stage to push them towards the online survey, while for the CATI mode there was no educational selectivity for receiving a reminder. Regarding the web survey mode, it is found that panellists having lower language proficiencies were more likely to get reminders. Panellists with personal traits such as persistence and decisiveness also did not need reminders animating them to start completing the questionnaire.

In sum, for a cognitive demanding mode such as the self-administered online questionnaire, reminders are particularly necessary for socially disadvantaged target persons, net of variables indicating the consequences of their social origin directly and indirectly. This is the reverse for the interviewer-administered mode, such as the CATI. This fact could explain the social homogeneity among the panellists who did not take part in the initial online mode in spite of receiving several reminders. However, it has to be analysed in future whether this result depends also on the sequence of self- and interviewer-administered modes.

Finally, it is worth noting that women, net of other influences, were less likely to get reminders than men. It is unsolved why there is a gender difference in the likelihood of receiving reminders in the CAWI and CATI modes. A simple answer would emphasize emphasise that women are more likely to take part at in our survey in the initial stages of the field period. Therefore, the next question arises: why is there a gender differentiation in survey participation? In this survey, interactions of respondents’ gender with their other characteristics which are not documented here did not dissolve the gender differentials. Thus, these gender-related facts should be investigated in future studies.

## Summary and conclusions

Since high and steady response rates are extremely important for longitudinal studies (Jäckle et al. 2015), the aim of this empirical contribution was to answer the question of whether sequential mixed-mode strategies are useful to realise such a goal. The main problem considered in this study is the question of what happens if nonrespondents are offered a second mode (interviewer-administered telephone interview) two weeks after survey launch due to their failure to respond to the first offered mode (self-administered web) when they are permitted to complete the online questionnaire even in the second stage of the field period. Since researchers investigating survey methodologies are interested in the effects of sequential designs in longitudinal studies (de Leeuw 2018), it should be investigated if this sequential offer, and the related decision, results in different patterns and a different social composition of survey participation across different stages of the field period. The research question behind this study was therefore: how many of the panellists, and which of them, take part in one of the offered survey modes, and at what point during the field period? To answer these questions, it has been taken into account that the effects of the “push-to-web” procedure, prepaid monetary incentives, and mode preference have been well-investigated (e.g. Becker et al. 2019; Dillman 2017; Singer and Ye 2013; Olson et al. 2012). However, in regard to panellists’ procrastination as regards survey response and nonresponse, it is also interesting to analyse the effect of reminders as an integral part of the TDM suggested by Dillman et al. (2014), and the question of who gets a reminder at what point in the field period. The second research question was therefore: what roles do follow-up reminders sent after the paper prenotification and invitation email play in improving the target persons’ willingness to participate in one of the offered survey modes? Are there different effects of reminders on survey participation depending on survey mode?

These questions were answered by using longitudinal survey paradata from a multiple-panel study on the educational and occupational trajectories of young people born around 1997 and living in the German-speaking cantons of Switzerland (Becker et al. 2020a). Focusing on the two most recent panel waves, methodological techniques and statistical procedures of event history analysis were utilised by considering competing risk models in particular. These models are necessary for analysing competing decisions at the same time, such as completing an online questionnaire or choosing a telephone interview.

Regarding response rates, the timing of survey participation, and features of survey management, the results were straightforward. Panellists who did not take the initially offered mode were more likely to use the subsequent mode. In this respect, it can be concluded that the sequential mixed-mode design works as intended. On the one hand, panellists who postpone their survey participation can be stimulated to take part at a more convenient point in time; on the other hand, the mode preference can be satisfied in this manner in a more efficient way than in concurrent mixed-mode designs. Furthermore, it was found, indirectly at least, that switching from one mode to another did not seem to result in the confusion of panellists at risk in regard to survey participation. The negative consequences of the theoretically assumed “paradox of choice” between two different survey modes versus nonresponse seem to be overrated. The careful application of the TDM—in particular, the use of reminders—seems to be useful to minimise the problems related with the offer of different survey modes at the same time. Finally, it was revealed that multiple reminders were indeed important for each of the subsequent modes, to animate panellists to take part in the survey. In the case of individuals postponing their participation it is assumed that reminders contribute to reducing the problem of cognitive overload due to the choice of a survey mode. However, in this study the impact of reminders faded in relation to their timing and number. The first reminders sent at early periods of each of the different modes were the most effective instruments; follow-up reminders sent later worked less well than the initial reminders. In sum, compared to the conventional sequential mixed-mode design, it was found for this panel study that the simultaneous offer of two different survey modes provided no significant advantages or disadvantages in terms of the amount, timing or selectivity of survey participation.

This empirical contribution has some limitations. The sample is limited to a single birth cohort and to a large spatial area in Switzerland. Individuals born around 1997 and living in the German-speaking cantons of Switzerland might be familiar with the internet (“digital natives”), and therefore the response rate for the web-based survey would be higher compared to the traditional telephone interview used as an additional mode. It is interesting to report that the share of target persons who use CATI has decreased from 40% in Wave 4 to 16% in Wave 7, and to 7% in the most recent Wave 8. Therefore, the findings reported above might not be generalisable to other more diverse samples, without any comparison with older and younger birth cohorts. Furthermore, the analysis is limited to the most recent seventh and eighth panel waves, conducted in the spring of 2018 and 2020. This means the panellists were experienced in the sequential mixed-mode design that had been utilised since the fourth panel wave in 2014.

Finally, talking generally about this type of surveys, it remains an open question if mode-specific biases counteract the benefits of minimising attrition across surveys, maximising the target persons’ response in each of the surveys, and reducing the latency in the field periods. Kreuter, Presser and Tourangeau (2008) found that social desirability bias is lower in the self-administered web mode than in the CATI mode, while the accuracy of respondents’ reports is higher in the online mode of data collection. According to their study, this is advantageous in surveys with a sequential mixed-mode design, when the response rate in the CAWI mode will be maximised while the minority of procrastinating interviewees will be caught by CATI, the second mode. In this way, the impact of mode-specific biases will be minimised. This procedure contributes to the collection of accurate longitudinal data on individuals’ educational and occupational trajectories. Whether or not this works in practice is something that must be investigated in the future.

## Data Availability

The data from the first seven waves of the panel study are available as Scientific Use Files at FORS in Lausanne and can be found in the online catalogue under the reference number 10773 (https://forsbase.unil.ch/project/study-public-overview/15802/0/). Data from Wave 8 will be available for the scientific community in 2021. The paradata from the fieldwork, as well as the time series data, can be requested from the author.

## Footnotes

1 According to Bianchi et al. (2017: 8), it could be possible that heterogeneity across modes in response propensities results in smaller compositional biases with mixed-mode designs than with single-mode designs. Using this survey management approach, both nonresponse and measurement error bias could be minimised (Sakshaug et al. 2019: 567).

2 They are measured in the first and second waves. *Persistency* is measured by the respondent’s agreement with the following five statements: “I do not like unfinished business.” “If I decide to accomplish something, I manage to see it through.”, “I complete whatever I start.”, “Even if I encounter difficulties, I persistently continue.”, and “I even keep at a painstaking task until I have carried it through”. The *control beliefs* are measured by six items indicating the respondent’s internal and external locus of control suggested by Jakoby and Jacob (1999): “I like to take on responsibility.”, “Making my own decisions instead of relying on fate has proved to be good for me.”, “In the case of problems and resistance, I generally find ways and means to assert myself.”, “Success depends on luck, not on performance.”, “I feel like having little influence on what happens to me.” and “When making important decisions, I lean on the behaviour of others”. *Decisiveness* is based on a question about the respondent’s decision certainty: “Life is full of decisions that need to be taken. Which of the six statements apply to you?” The wordings of these statements are: “I am really unsure as to what I should decide and often waver back and forth.”, “Others unsettle me in my decision.”, “After making a decision, I have great doubts as to whether I really made the right decision.”, “It is very hard for me to decide because there are so many possibilities.”, “When I make a decision, I stick to it.”, “I am convinced that sooner or later I will make an occupational decision that is right for me”. For each of these items, the agreement itself consists of a scale of discrete values from 1 for “I strongly disagree” to 5 for “I strongly agree”. In order to reduce complexity and to avoid multicollinearity, three factors – persistency, control beliefs and decisiveness – are extracted by confirmatory factor analysis.

3 One the one hand, the rate of participation in the online mode increased across the consecutive surveys, while the latency decreased after the fourth wave (Becker and Glauser 2018; Becker et al. 2019: 227). On the other hand, the singular effect of a public shutdown related to the COVID-19 pandemic, which had been going on for almost two months before survey launch and was still occurring in the first 10 days after the start of the fieldwork in Wave 8, was detected (Becker et al. 2020b). In the setting of a “natural experiment” for the first 10 days of fieldwork after survey launch, the non-pharmaceutical official orders and arrangements in the course of the COVID-19 pandemic resulted in a significantly higher response rate and shorter latency compared to Wave 7.

## Code availability

Each of the software applications that was used is part of the Stata package. The custom code can be requested from the author.
